# Accurate Structures and Spectroscopic Parameters of Guanine Tautomers in the Gas Phase by the Pisa Conventional and Explicitly Correlated Composite Schemes (PCS and PCS-F12)

**DOI:** 10.1021/acs.jpca.3c03999

**Published:** 2023-08-03

**Authors:** Vincenzo Barone, Silvia Di Grande, Federico Lazzari, Marco Mendolicchio

**Affiliations:** † Scuola Normale Superiore, Piazza dei Cavalieri 7, Pisa 56126, Italy; ‡ Scuola Superiore Meridionale, Largo San Marcellino 10, Napoli 80138, Italy

## Abstract

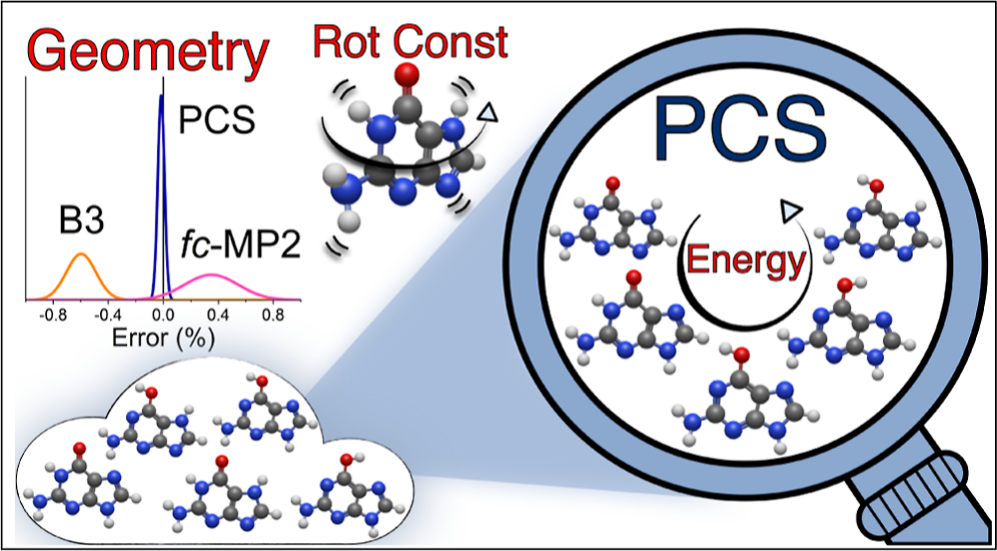

A general strategy for the accurate computation of structural and spectroscopic properties of biomolecule building blocks in the gas phase is proposed and validated for tautomeric equilibria. The main features of the new model are the inclusion of core-valence correlation in geometry optimizations by a double hybrid functional and the systematic use of wave-function composite methods in conjunction with cc-pV*n*Z-F12 basis sets with separate extrapolation of MP2 and post-MP2 contributions. The resulting Pisa composite scheme employing conventional (PCS) or explicitly correlated (PCS-F12) approaches is applied to the challenging problem of guanine tautomers in the gas phase. The results are in remarkable agreement with the experimental structures, relative stabilities, and spectroscopic signatures of different tautomers. The accuracy of the results obtained at reasonable cost by means of black-box parameter-free approaches paves the way toward systematic investigations of other molecular bricks of life also by non-specialists.

## 1 Introduction

Most molecular bricks of life (amino acids, nucleobases, sugars, etc.) undergo either conformational or tautomeric equilibria, which are tuned by both intrinsic stereo-electronic and environmental effects. An unbiased analysis of the role played by the different contributions on the overall experimental outcome calls for a preliminary investigation of gas phase processes. We have recently devised and validated a general computational strategy able to disentangle the complex conformational landscapes of amino acids.^1,2^ Here, we tackle the problem of tautomeric equilibria, which involve bond pattern changes and are, therefore, more challenging for quantum chemical (QC) computations.

Among all possible tautomeric forms of the nucleobases, the so called “canonical” (keto and amino) forms predominate over their “minor” enol and imino counterparts under physiological conditions. In the case of uracil, thymine, and adenine, the “canonical” tautomer is significantly more stable than all the “minor” ones also in the gas phase, whereas the situation is more involved for cytosine and guanine.^3^ The tautomeric equilibrium of cytosine has been recently investigated by state-of-the-art QC methods.^4,5^ Therefore, the focus of the present work is on guanine.

From the theoretical point of view, most QC calculations predict that there are small energy differences between the lowest energy tautomers of guanine, with the quantitative values being extremely sensitive to the level of theory.^6−8^ However, even the most refined computations performed until now employed geometrical structures and force fields of limited accuracy, thus compromising any unbiased comparison with the results of high-resolution spectroscopy. Furthermore, zero-point energies (ZPEs) have been computed within the rigid-rotor harmonic-oscillator (RRHO) model with low-level quantum chemical methods.

In our opinion, the most effective computational strategy for this kind of problems is obtained by combining different QC methods for a preliminary exploration of potential energy surfaces (PESs) and the successive refinement of the most significant stationary points.^9−11^ In this framework, once a suitable panel of low-energy minima has been identified, accurate structures^12,13^ and relative energies must be computed.^14−19^ Finally, ZPEs and spectroscopic parameters of the energy minima, with non-negligible populations under the experimental conditions of interest, are obtained.^20^

In this connection, for systems not showing strong multi-reference character, the coupled cluster (CC) ansatz including single, double, and a perturbative estimation of triple-excitations [CCSD(T)]^21^ is considered the *gold standard* of contemporary computational chemistry, provided that complete basis set (CBS) extrapolation and core-valence (CV) correlation are taken into account.^22^ Based on this premise, we have developed in the last years an effective composite method, referred to as the *cheap* scheme (ChS), which delivers accurate energies at a reasonable cost thanks to the evaluation of CBS extrapolation and CV correlation using second-order Møller–Plesset perturbation theory (MP2^23^), starting from CCSD(T) computations in conjunction with a triple-zeta basis set. Several benchmarks have shown that, without the need for any empirical parameter, the ChS model closely approaches the accuracy of the corresponding (much more costly) scheme in which CBS and CV contributions are evaluated at the CCSD(T) level.^14,24,25^ More recently, improved versions employing the *june* partially augmented basis sets^26^ (junChS)^10,15^ and replacing the conventional post-Hartree-Hock contributions by explicitly correlated^27^ (F12) approaches (junChS-F12)^16,28,29^ have been developed. Thanks to these improvements, the junChS and junChS-F12 models provide accurate results for a large panel of properties including geometrical structures, thermochemical and kinetic parameters, vibrational frequencies and non-covalent interactions.^1,30^ Here, we take a step further, employing the conventional and explicitly correlated versions of the new, more accurate, Pisa composite scheme (PCS and PCS-F12, respectively) described in the next section.

The present work is devoted to the study of the relative stabilities and high-resolution spectra of the four species of guanine detected in the gas phase by microwave (MW) spectroscopy.^7^ As already mentioned, previous computational studies of this molecule employed QC methods of limited accuracy or payed marginal attention to the geometrical and vibrational parameters. However, an *a priori* prediction of the spectroscopic outcome requires the simultaneous calculation of accurate structures, relative stabilities, and spectroscopic parameters. In the following sections, it will be shown that the integrated computational strategy sketched above paves the way toward the systematic achievement of this task for the main molecular bricks of life by a fully unsupervised tool, which can be routinely employed also by non-specialists.

## 2 Methods

On the basis of previous experience, a first characterization of PESs is performed at the B3LYP/6-31+G* level,^31,32^ also including Grimme’s D3BJ dispersion corrections.^33^ This combination of functional and basis set, which is also used for the computation of anharmonic contributions, will be referred to in the following as B3/SVP. Next, the geometries of the most stable species are refined at levels of theory sufficiently accurate to allow a direct comparison with the leading terms of MW spectra, namely, rotational constants of the vibrational ground state (*B*_τ_^0^, where τ refers to the inertial axes *a*, *b*, *c*). In the framework of second-order vibrational perturbation theory (VPT2),^34−38^ each *B*_τ_^0^ can be split into an equilibrium contribution (B_τ_^eq^) and a vibrational correction (Δ*B*_τ_^vib^), with the latter term including contributions from harmonic force constants, Coriolis couplings and, above all, semi-diagonal cubic force constants.^39,40^ The Δ*B*_τ_^vib^ terms are typically smaller than 1% of the corresponding *B*_τ_^eq^ rotational constants,^41^ so that errors of the order of 10% (well within the typical accuracy of B3/SVP computations) are acceptable.^40,42^ However, the needed accuracy for equilibrium rotational constants (0.1–0.2%) can be reached only employing state-of-the-art QC methods.^43,44^

In previous works, we employed the rev-DSD-PBEP86-D3BJ functional^45^ (hereafter rDSD) in conjunction with a partially augmented triple-zeta basis set (jun-cc-pVTZ,^26^ hereafter j3). The systematic nature of the errors of this model permits to improve significantly the rDSD/j3 geometrical parameters by a linear regression (LR) approach.^13^ Even better results can be obtained resorting, when possible, to templating molecules (TMs) sharing structural similarities with the species under study and whose accurate equilibrium structures are already available.^13^ The resulting model (referred to as nano-LEGO^13^ or LEGO-Bricks^46^) has met with considerable success, but suffers from some limitations, mainly related to the presence of several empirical parameters in the LR approach and the limited number of available accurate structures for the fragments to be employed as TMs.

Systematic investigations^47^ showed that the use of empirical parameters can be avoided by combining CV correlation computed at the MP2 level in conjunction with the cc-pwCVTZ basis set (hereafter wC3)^48^ and valence contributions computed at the rDSD level in conjunction with the cc-pVTZ-F12 basis set (hereafter 3F12).^49^ These choices lead to the first component of the new Pisa composite schemes (PCS), in which each geometrical parameter *r* is obtained combining the corresponding parameters optimized at different levels
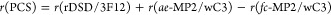
1where *ae* and *fc* stand for all-electron and frozen-core, respectively. The accuracy of PCS geometrical parameters will be compared to that of several other approaches with reference to the tautomers of guanine detected in the gas phase.

In addition to structural parameters, accurate electronic energies are needed to determine the relative abundance of low-energy species. For this purpose, in the last few years, we have systematically employed the junChS and junChS-F12 models.^1,30^ However, some aspects of these approaches can be further improved, enhancing the accuracy of the final results without any excessive increase of computational resources. The starting point of the new conventional PCS model is a *fc*-CCSD(T) calculation in conjunction with the 3F12 basis set, already employed for geometry optimizations. Next, the CV correlation is obtained at the MP2 level exactly in the same way as for geometrical parameters. Finally, the CBS extrapolation is performed employing the 3F12 and cc-pVQZ-F12 (hereafter 4F12)^49^ basis sets for the MP2 contribution, whereas the cc-pVDZ-F12 (hereafter 2F12)^49^ and 3F12 basis sets are employed for the difference between CCSD(T) and MP2 energies. Both CBS extrapolations are performed by the standard *n*^–3^ two-point formula.^50^ The final PCS energy can be written as follows
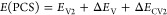
2where
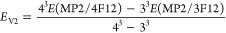
3and
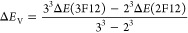
4with
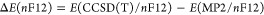
5Finally
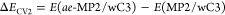
6In the equations mentioned above, all the energies have been obtained employing the *fc* approximation, unless the label *ae* is explicitly employed. The availability of the reduced cost FNO-CCSD(T) implementation^17^ (not used in the present context) paves the way toward the systematic study of large-sized molecules at this level. It is to be noted that the junChS model^10,15^ is recovered when the jun-cc-pV*n*Z basis sets^26^ (hereafter j*n*) are used in place of their *n*F12 counterparts and Δ*E*_V_ = *E*(CCSD(T)/jun-cc-pVTZ) – *E*(MP2/jun-cc-pVTZ).

Replacement of the conventional methods in the evaluation of the *E*_V2_ and Δ*E*_V_ contributions by their explicitly-correlated counterparts in conjunction with the same basis sets leads to the PCS-F12 version. The advantage of this model is that the role (hence the incertitude) of the CBS extrapolation is strongly reduced without any excessive increase of the computational resources. In particular, use of the accurate and size-consistent CCSD(F12*)(T+) model^18^ increases the robustness of the approach^29^ and the availability of the reduced cost FNO-CCSD(F12*)(T+) version^51^ (not used in the present context) allows the study of large systems. Scalar relativistic contributions and diagonal Born-Oppenheimer corrections (DBOC) can be added when needed,^10,30^ but previous computations showed that they play a negligible role on the relative stability of different tautomers.^8^ ZPEs are usually obtained within the RRHO approximation, possibly employing empirical scale factors.^52^ In the present context, the use of empirical factors is avoided by resorting to a resonance-free VPT2 expression^53−56^ and employing rDSD/3F12 harmonic frequencies combined with B3/SVP anharmonic contributions. All the density functional theory (DFT) and conventional wave-function computations have been performed with the Gaussian package,^57^ whereas all the explicitly correlated ones have been performed with the aid of the MRCC^58,59^ software.

## 3 Results and Discussion

**Figure 1:**
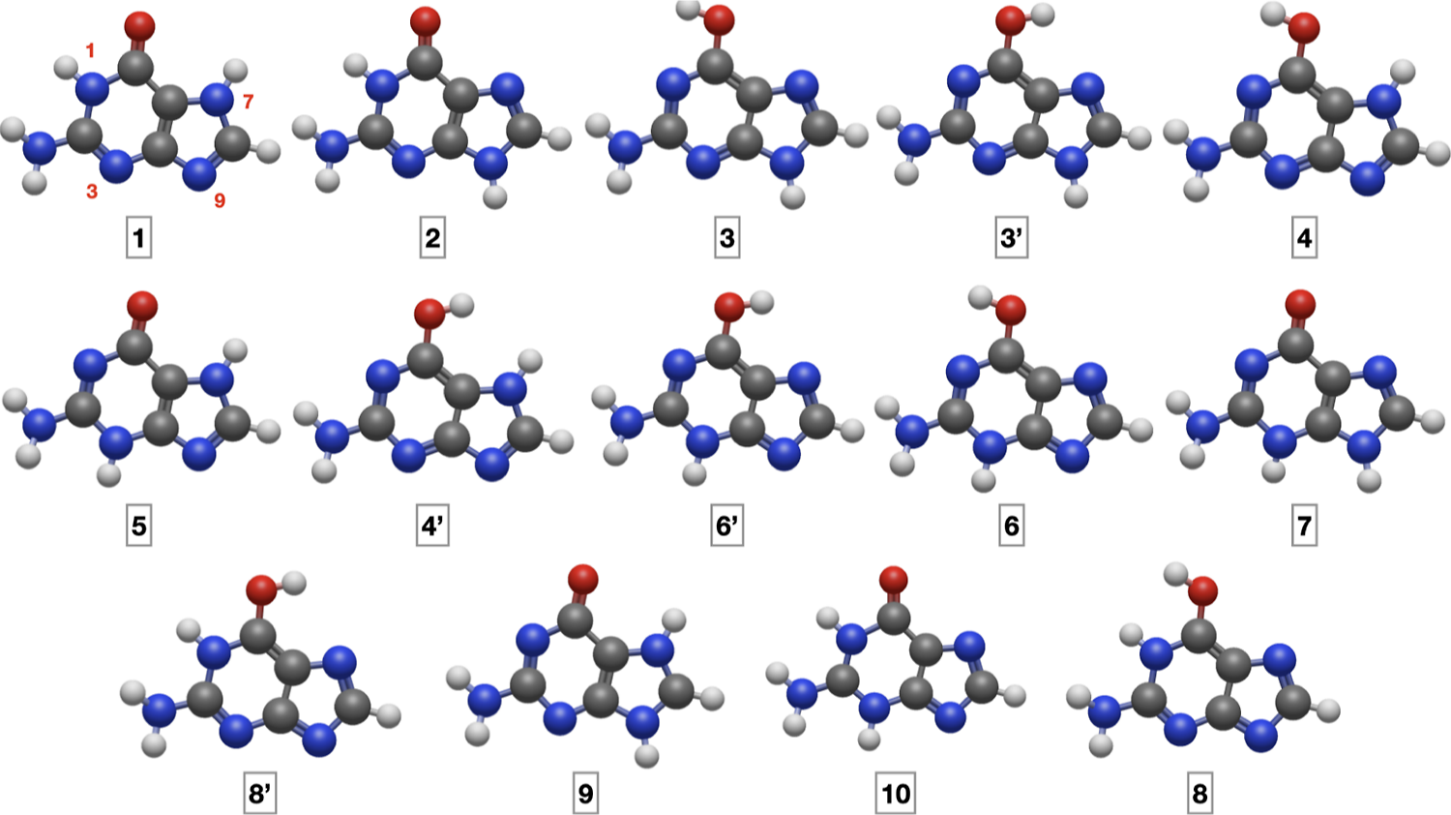
Structure of the amine tautomers and rotamers of guanine.

The number of possible tautomers (*N*_T_) of a given species is *N*_T_ = *N*_S_!/[*N*_H_!(*N*_S_ – *N*_H_)!], where *N*_S_ is the number of tautomeric sites and *N*_H_ the number of labile protons. Guanine has 4 endo (N1, N3, N7, N9) and 2 exo (O=C and NH_2_) tautomeric sites and 3 labile protons, so that *N*_S_ = 6, *N*_H_ = 3, and *N*_T_ = 20. Among those tautomers, there are 10 amino and 10 imino species. All the amino species are shown in Figure 1 in order of decreasing stability (according to junChS-F12 electronic energies, vide infra) and their numbering follows that of ref (8). Two keto-amino (KA) and one enol-amino (EA) tautomers are possible for each of the two non-equivalent structures of the imidazole ring (N7H and N9H), namely, the KA structures **1** and **5** together with the EA structure **4** in the former case and the KA structures **2** and **7** together with the EA structure **3** in the latter case. Furthermore, both N7 and N8 can be protonated or deprotonated at the same time. In the first case, only the KA form **9** is possible, whereas in the latter case, one KA (**10**) and two EA (**6**, **8**) forms are possible. Finally, two rotamers are possible for each EA tautomer (**3**, **3’**; **4**, **4’**; **6**, **6’;** and **8**, **8’**). When needed, the different species will be indicated by two letters (KA and EA for keto and enol tautomers, respectively), followed by one (for EA forms) or two (for the KA forms) numbers indicating the positions of the other two acidic hydrogens.

For instance, the species **1**, **2**, **3,** and **4** will be labeled KA17, KA19, EA9, and EA7, respectively. Finally, the *cis* arrangement of the enol hydrogen with respect to N7 will be indicated by a *c* subscript (e.g., species **3’** is EA_*c*_9). For imino species, the *cis* arrangement of the imino hydrogen with respect to N1 will be indicated by another *c* subscript and up to three numbers are used to define the positions of acidic hydrogens for KI tautomers. In analogy with the case of cytosine,^5^ the equilibrium structures of imino tautomers are planar (*C*_s_ symmetry), whereas the amino tautomers are slightly non-planar (*C*_1_ symmetry), in agreement with the negative values of the inertial defects (Δ = *I*_*c*_ – *I*_*a*_ – *I*_*b*_) derived for some of them from the experimental microwave spectra.^7^

As already mentioned in the methods section, preliminary B3/SVP optimized geometries are refined at the rDSD/j3 level. On top of these latter geometries, improved energies are obtained by single-point computations at the MP2-F12/j3, CCSD(F12*)(T+)/j3 and junChS-F12 levels. The relative stability of all the amino rotamers and tautomers of guanine computed by these methods are collected in Table 1.

**Table 1:** Relative Electronic Energies of All the Tautomers and Rotamers of Guanine Computed by Different Methods and Relative Harmonic B3/SVP Zero-Point Energies (ΔZPE)^a^

tautomer	B3/SVP	rDSD/j3	MP2-F12/j3	CC-F12/j3^b^	junChS-F12	ΔZPE^c^
**1**	0.0	0.0	0.0	0.0	0.0	0.0
**2**	2.9	2.5	3.1	2.8	2.9	–0.4
**3**	12.4	6.5	3.5	3.0	3.0	–0.6
**3’**	15.0	7.6	4.1	4.2	4.0	–0.7
**4**	24.4	18.8	15.2	15.0	14.9	–1.3
**5**	27.7	25.6	25.2	26.1	25.7	–0.7
**4’**	59.2	49.6	46.6	45.7	45.6	–4.1
**6’**	63.7	55.6	51.6	53.2	53.3	–0.6
**6**	72.0	64.2	61.3	61.9	62.2	–1.1
**7**	84.8	79.9	81.6	80.0	79.9	–2.9
**8’**	95.5	86.4	84.6	83.1	83.6	–1.6
**9**	81.9	84.4	82.6	86.6	86.4	–1.1
**10**	102.7	100.4	97.7	102.6	102.9	–3.5
**8**	139.3	127.3	127.5	123.7	124.5	–4.6

a All the energy evaluations have been performed on top of rDSD/j3 geometries, except B3/SVP computations, which employ B3/SVP geometries.

b CC-F12 stands for CCSD(F12*)(T+).

c From harmonic B3/SVP frequencies. All the values are given in kJ mol^–1^.

Taking the junChS-F12 results as references, the only inversion in the stability order provided by the other methods concerns species **8’** and **9** (rDSD and MP2-F12) and **7** (B3/SVP). Furthermore, both rDSD and MP2-F12 models perform quite a good job, with a maximum error of 4 and 5 kJ mol^–1^, respectively. However, the rDSD model underestimates systematically the stability of the enol tautomers by more than 3 kJ mol^–1^, with this value corresponding to a relative error close to 50% for the species **3** and **3’**. The errors are more evenly distributed in the case of the MP2-F12 model, which can be, therefore, used for qualitative analyses. Exploratory computations showed that, contrary to the case of cytosine,^5^ all the imino species are considerably less stable than the corresponding amino forms. Just to give an example, the EI_*c*_19 form lies about 100 kJ mol^–1^ above the most stable tautomer (KA17 (**1**)) according to all the employed methods. As a consequence, imino tautomers will not be considered anymore. Furthermore, all the methods confirm that only four species [KA17 (**1**), KA19 (**2**), EA9 (**3**), and EA_*c*_9 (**3’**)] should have non-negligible populations in the gas phase. Therefore, in the following, the attention will be focused on these species together with EA7 (**4**), which has not been detected in the most recent microwave study,^7^ but should be not much less stable.

The PCS equilibrium geometries of all the guanine tautomers and rotamers are given in the Electronic Supporting Information, whereas the equilibrium rotational constants of the five most stable forms computed at different levels are collected in Table 3. In the same table are also given the semi-experimental (SE)^60,61^ rotational constants obtained from the experimental ground state values reported in ref (7) and the B3/SVP vibrational corrections given in Table 2. It is quite apparent that the B3LYP and MP2 computations routinely employed in the interpretation of MW spectra can provide at most qualitative trends and that at these levels, the computation of vibrational corrections is not warranted. Already rDSD/j3 computations perform a better job, and correction of bond lengths by the LR approach further improves the accuracy. However, thanks to both extension of the basis set and inclusion of CV correlation (which play comparable roles), the PCS results are even more accurate, without the need of any empirical parameter in addition to those possibly present in the underlying electronic structure method.

**Table 2:** Equilibrium Rotational Constants and Vibrational Corrections for the Five Most Stable Energy Minima of Guanine Computed at the B3/SVP Level^a^

	*B*_*a*_^eq^	*B*_*b*_^eq^	*B*_*c*_^eq^	Δ*B*_*a*_^vib^	Δ*B*_*b*_^vib^	Δ*B*_*c*_^vib^
**1**	1910.4	1115.1	704.7	–11.6	–6.7	–4.2
**2**	1911.5	1109.4	702.4	–12.0	–6.5	–4.2
**3**	1908.6	1124.5	707.0	–13.3	–6.0	–4.2
**3’**	1916.0	1128.3	710.3	–13.8	–6.1	–4.3
**4**	1912.0	1135.9	712.8	–12.7	–6.2	–4.2

a All the values are given in MHz.

**Table 3:** Comparison between SE Equilibrium Rotational Constants and the Equilibrium Rotational Constants Obtained by Different QC Methods for the Five Most Stable Tautomers and Rotamers of Guanine Obtained by Different Methods^a^

	parameter	SE^b^	rDSD/j3	LR	rDSD/3F12	PCS	MP2/wC3	*ae*-MP2/wC3
**1**	B_*a*_^eq^	1933.8	1923.2	1932.0	1927.0	1934.1	1921.3	1928.5
	B_*b*_^eq^	1128.4	1122.8	1128.0	1124.5	1127.9	1128.6	1132.0
	B_*c*_^eq^	713.2	709.5	712.8	710.7	713.0	711.7	714.1
	MUE		6.6	0.9	4.4	0.3	4.7	3.3
	MAX		10.6	1.8	6.8	0.4	12.5	5.3
	MUE %		0.52	0.06	0.35	0.03	0.29	0.24
	MAX %		0.55	0.09	0.35	0.04	0.64	0.32
**2**	B_*a*_^eq^	1934.3	1923.6	1932.5	1927.3	1934.0	1922.3	1929.1
	B_*b*_^eq^	1123.2	1117.4	1122.5	1119.1	1122.7	1122.9	1126.4
	B_*c*_^eq^	711.1	707.3	710.5	708.5	710.8	709.5	711.8
	MUE		6.7	1.0	4.5	0.3	4.6	3.0
	MAX		10.7	1.8	7.0	0.5	12.0	5.2
	MUE %		0.53	0.07	0.36	0.03	0.29	0.22
	MAX %		0.55	0.09	0.36	0.05	0.62	0.29
**3**	B_*a*_^eq^	1929.4	1920.0	1928.9	1923.2	1929.7	1921.6	1928.1
	B_*b*_^eq^	1138.4	1132.9	1138.1	1134.7	1138.3	1137.3	1141.0
	B_*c*_^eq^	716.4	712.8	716.0	713.9	716.2	714.9	717.2
	MUE		6.2	0.4	4.1	0.2	3.4	1.6
	MAX		9.4	0.5	6.2	0.3	7.8	2.6
	MUE %		0.49	0.03	0.33	0.01	0.24	0.14
	MAX %		0.51	0.05	0.35	0.02	0.40	0.23
**3’**	B_*a*_^eq^	1937.3	1927.8	1936.8	1931.2	1937.7	1930.3	1937.0
	B_*b*_^eq^	1142.1	1136.7	1141.9	1138.5	1142.1	1141.3	1145.0
	B_*c*_^eq^	719.0	715.3	718.6	716.5	718.8	717.6	719.9
	MUE		6.2	0.4	4.1	0.2	3.1	1.3
	MAX		9.4	0.5	6.1	0.4	7.0	2.9
	MUE %		0.49	0.03	0.33	0.02	0.21	0.13
	MAX %		0.51	0.05	0.35	0.03	0.36	0.25
**4**	B_*a*_^eq^		1909.3	1918.2	1912.9	1919.5	1909.9	1916.5
	B_*b*_^eq^		1135.4	1140.6	1137.2	1140.7	1139.8	1143.2
	B_*c*_^eq^		712.3	715.6	713.5	715.8	714.2	716.5

a All the values (except % errors) are given in MHz.

b SE equilibrium rotational constants obtained from the experimental ground state rotational constants of ref (7) and the B3/SVP vibrational corrections of Table 2.

**Figure 2:**
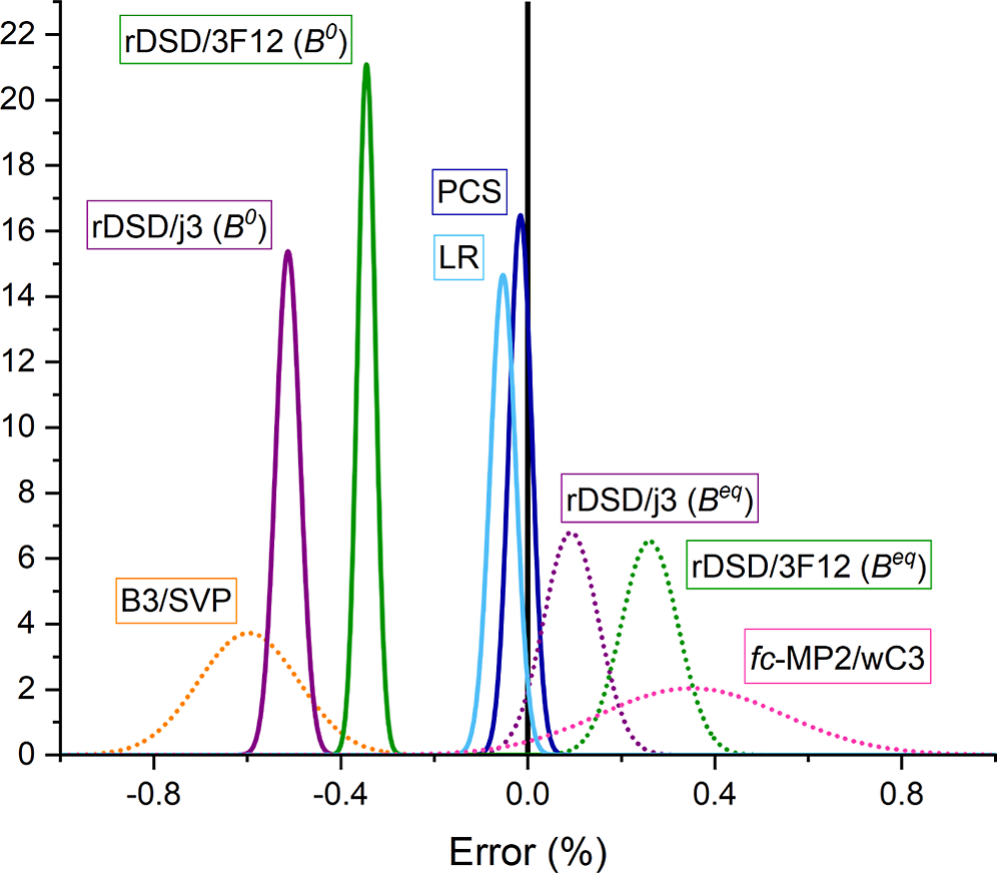
Relative (%) deviations of computed rotational constants from the reference experimental values.

As a matter of fact, the PCS relative mean unsigned error (MUE %) is always close to 0.02% and the corresponding relative maximum unsigned error (MAX %) never exceeds 0.05%, with these values being on par with the results delivered by the most sophisticated (and much more expensive) wave-function composite methods for small semi-rigid molecules.^41,43^ All these trends can be better appreciated by the normalized error statistics drawn in Figure 2.

The contribution of CV correlation, whose main effect is to shorten bond lengths (hence to augment rotational constants), increases with the atomic number of the involved atoms: as a consequence, its effect is smaller for X–H than for X–Y bonds (with X, Y being second- or third-row atoms). Also, the role of vibrational corrections is especially important for bond lengths, but the leading term (related to cubic force constants) produces ground state bonds that are longer than their equilibrium counterparts, with the X–H distances being especially affected due to their strong anharmonicity. Therefore, the inclusion of vibrational corrections would further decrease the already underestimated B3LYP rotational constants and the inclusion of CV correlation would further increase the already overestimated MP2 rotational constants. On the other hand, the balanced treatment of both contributions improves the already satisfactory rDSD/3F12 results. Actually, the errors on equilibrium rotational constants obtained at the PCS level are close to the expected errors of vibrational corrections, so that we are approaching the intrinsic accuracy limit of structure determinations by QC methods.

Guanine contains five ^14^N nuclei with spin *I* = 1 and with a nuclear quadrupole moment that couples to the molecular-electric-field gradient at the site of the nuclei, with this causing the coupling of the nuclear spin to the overall rotational momentum. The presence of five N nuclei results in very complex hyperfine splitting patterns for all observed rotational lines. While the corresponding quadrupole coupling constants can be computed quite accurately at the rDSD level,^1^ they do not provide any additional information since no attempt was made in the experimental studies to assign the quadrupole hyperfine components, and the rotational frequencies were measured as the intensity-weighted mean of the line clusters.^7^ In the same vein, the applied microwave power for optimal polarization of the rotational transitions was consistent with the predicted values of the electric dipole moment components for each tautomer.

The results collected in Table 4 for the five most stable species show that, contrary to the case of geometrical parameters, CV correlation plays a negligible role in tuning the stability of different species. As expected, rDSD/j3 optimized geometries are already sufficiently reliable for the computation of accurate electronic energies: for instance, the average difference between CCSD(T)/3F12 relative energies at rDSD/j3 and PCS geometries is within 0.1 kJ mol^–1^. The PCS and PCS-F12 results are in good agreement with the maximum and average difference between the relative stabilities predicted by the two methods being 0.3 and 0.2 kJ mol^–1^, respectively. Actually, also the junChS-F12 results (which employ rDSD/j3 geometries) are sufficiently accurate (see Table 1), with maximum and average difference of 0.2 and 0.1 kJ mol^–1^ from PCS (0.4 and 0.3 kJ mol^–1^ from PCS-F12). This result gives further support to the reliability of the relative stabilities given in Table 1 for all the amine tautomers and rotamers of guanine. It is also noteworthy that the PCS-F12 relative stabilities are virtually identical (maximum difference of 0.1 kJ mol^–1^) to the best computations performed until now (W1-F12),^8^ despite the use of quite different equilibrium geometries (PCS in the present case and B3LYP in ref (8)). Finally, the main effect of ZPEs is to increase the relative stability of all the other tautomers (and rotamers) with respect to the KA17 (**1**) species by about 1 kJ mol^–1^. It is remarkable that the contributions to the relative stabilities of our anharmonic ZPEs and the scaled harmonic ones employed in ref (8) show differences (maximum 0.7 and average 0.5 kJ mol^–1^) larger than those between electronic energy contributions. This result confirms the importance of employing refined vibrational contributions for obtaining accurate thermochemical data. On the other hand, all the computational approaches agree with the semi-quantitative experimental estimates of relative stabilities derived from the intensities of MW signals^7^ and the infrared results with He droplets.^62^ In fact, all the methods forecast a larger population of KA tautomers [KA17 (**1**) and KA19 (**2**)] with respect to EA tautomers [EA9 (**3**) and E_*c*_A9 (**3’**)].

**Table 4:** PCS and PCS-F12 Relative Stabilities of the Most Stable Guanine Tautomers, together with the Main Contributions to the Overall Results (in kJ mol^–1^)

tautomer	Δ*E*^V2^	Δ*E*^V2*F*12^	Δ(Δ*E*^CV2^)	Δ(Δ*E*^V^)	Δ(Δ*E*^VF12^)	Δ*E*^PCS^	Δ*E*^PCS-F12^	ΔZPE^a^
**1**	0.0	0.0	0.0	0.0	0.0	0.0	0.0	0.0
**2**	3.2	3.2	0.0	–0.4	–0.4	2.8	2.8	–0.7
**3**	3.1	3.5	0.0	–0.0	–0.2	3.1	3.3	–1.1
**3’**	3.6	4.1	–0.1	0.6	0.4	4.1	4.4	–0.9
**4**	14.7	15.1	0.0	0.4	0.2	15.1	15.3	–1.6

a From rDSD/3F12 harmonic frequencies and B3/SVP anharmonic contributions. See main text for details.

## 4 Concluding Remarks

In this paper, a general strategy aimed at the *a priori* computation of accurate spectroscopic parameters for biomolecule building blocks has been further improved and applied to the challenging playground of the guanine tautomeric equilibrium in the gas phase. Accurate structures and relative energies are obtained by two new parameter-free composite schemes (PCS and PCS-F12), which employ very accurate molecular structures obtained at moderate cost by combining DFT (double hybrid) valence contributions with MP2 core-valence correlation. On top of these geometries, electronic energies are obtained by conventional or explicitly correlated models in conjunction with accurate yet effective recipes for CBS extrapolation, perturbative evaluation of contributions from triple excitations, and core-valence correlation. The results obtained for guanine are in full agreement with the available spectroscopic data and permit their unbiased interpretation in terms of the cooperation or competition between different stereo-electronic effects.

In a more general perspective, work is already in progress in order to further extend the dimensions of tractable systems by employing local correlation methods like PNO or DLPNO.^63−65^ Even pending those further developments, the results of the present investigation pave the way toward highly reliable investigations of structural and spectroscopic features for molecular bricks of life, possibly tuned by tautomeric equilibria, with the aid of fully unsupervised methods coupling accuracy and feasibility.

## Supporting Information Available

Cartesian coordinates of the PCS geometries for all the guanine tautomers (PDF)
