# Supplementary material for: Accurate Structures and Spectroscopic Parameters of Guanine Tautomers in the Gas Phase by the Pisa Conventional and Explicitly Correlated Composite Schemes (PCS and PCS-F12)
Source: J Phys Chem A. 2023 Aug 3;127(32):6771–8. doi: 10.1021/acs.jpca.3c03999 (PMC10440789; doi:10.1021/acs.jpca.3c03999)
Supplement: Supplementary file 1 — jp3c03999_si_001.pdf [file jp3c03999_si_001.pdf]

Supporting Information: Accurate Structures and Spectroscopic Parameters of Guanine Tautomers in the Gas Phase by the Pisa Conventional and Explicitly-Correlated Composite Schemes (PCS and PCS-F12)

Vincenzo Barone,*,† Silvia di Grande,†,‡ Federico Lazzari,† and Marco Mendolicchio†

†*Scuola Normale Superiore, Piazza dei Cavalieri 7, 56126 Pisa, Italy*

‡*Scuola Superiore Meridionale, Largo San Marcellino 10, 80138 Napoli, Italy*

E-mail: vincenzo.barone@sns.it

PCS geometries of the guanine tautomers

1

```
16

C -1.678913 -0.532847 -0.002959
N -0.748102 -1.433718  0.007835
C  0.517857 -0.923038 -0.000006
C  0.814243  0.430215  0.010707
C -0.189879  1.438188  0.003238
N -1.453128  0.822786 -0.004723
N  2.182197  0.513563  0.008744
N  1.670331 -1.660602 -0.005598
C  2.636594 -0.766139 -0.001102
O -0.069383  2.651693 -0.004911
N -3.001135 -0.909637 -0.072129
H -3.119112 -1.898538  0.082851
H -3.658586 -0.342994  0.438876
H  3.689989 -0.995590 -0.004316
H -2.232600  1.458598 -0.087500
H  2.724830  1.359950  0.011206
```

2

```
16

C -1.660781 -0.568130 -0.003300
N -0.691065 -1.437505  0.006890
C  0.529528 -0.844123 -0.000364
C  0.848601  0.503069  0.007338
C -0.218341  1.463108  0.002825
N -1.469746  0.781521 -0.003501
N  2.215197  0.684518  0.007312
N  1.727607 -1.494446 -0.003348
C  2.704395 -0.525420  0.000960
O -0.199631  2.674350 -0.002862
N -2.960373 -1.001790 -0.068764
H -3.054915 -1.988120  0.110300
H -3.658209 -0.432642  0.380385
H  3.752566 -0.776823 -0.000757
H -2.266349  1.397643 -0.072097
H  1.852213 -2.491972 -0.009816
```

3

16

```
C -1.654990 -0.568528 -0.005515
N -0.669969 -1.464825  0.002732
C  0.527869 -0.884071  0.000113
C  0.807296  0.483165 -0.003791
C -0.322819  1.303222 -0.000661
N -1.534289  0.777056 -0.000809
N  2.172178  0.713629 -0.002566
N  1.752874 -1.489591  0.004407
C  2.693073 -0.480938  0.001569
O -0.207963  2.631551  0.005660
N -2.932597 -1.044919 -0.059408
H -3.056983 -2.010634  0.186235
H -3.659777 -0.403069  0.199537
H  3.749566 -0.697195  0.002783
H -1.108436  2.982631  0.001855
H  1.919386 -2.480700  0.003524
```

3'

16

```
C -1.695960 -0.487603 -0.005085
N -0.757223 -1.438611  0.003438
C  0.466646 -0.918245 -0.000256
C  0.794353  0.431835 -0.005780
C -0.288837  1.314187 -0.001259
N -1.519732  0.853462 -0.000589
N  2.163643  0.623880 -0.003933
N  1.674843 -1.563476  0.004968
C  2.648502 -0.588770  0.001579
O -0.125143  2.641205  0.006805
N -2.992896 -0.901090 -0.055839
H -3.171555 -1.860304  0.179538
H -3.690241 -0.218229  0.178737
H  3.697129 -0.839558  0.003393
H  1.812994 -2.559237  0.005800
H  0.824145  2.820110  0.006587
```

4

```
16

C -1.675568 -0.535804 -0.005733
N -0.722137 -1.455736  0.003312
C  0.516080 -0.948726  0.000014
C  0.785088  0.423221 -0.006291
C -0.297464  1.290441 -0.000822
N -1.520843  0.813953 -0.000241
N  2.156601  0.537741 -0.003982
N  1.696519 -1.657328  0.006730
C  2.632501 -0.738925  0.003136
O -0.106375  2.619957  0.006941
N -2.970861 -0.970209 -0.063096
H -3.115742 -1.933609  0.182448
H -3.674573 -0.313142  0.221278
H  3.692067 -0.941720  0.005131
H -0.984634  3.023171  0.003834
H  2.695107  1.385446 -0.009105
```

4’

```
16

C -1.696413 -0.495212 -0.005750
N -0.763378 -1.439462  0.006009
C  0.480398 -0.957778  0.001112
C  0.780643  0.409791 -0.009364
C -0.287999  1.303916 -0.001313
N -1.514266  0.846790 -0.000786
N  2.162114  0.482544 -0.006282
N  1.643187 -1.697948  0.009735
C  2.602136 -0.809451  0.002607
O -0.161112  2.648398  0.009570
N -2.998276 -0.901043 -0.061707
H -3.172221 -1.860547  0.178113
H -3.690295 -0.221388  0.196346
H  3.656251 -1.038907  0.003261
H  2.749345  1.296139 -0.027162
H  0.767561  2.893761  0.020349
```

5

16

C	-1.735248	-0.358733	-0.001154
N	-0.770567	-1.329795	0.007592
C	0.532941	-0.902352	0.004511
C	0.800199	0.438237	0.011723
C	-0.230103	1.451466	0.000777
N	-1.521598	0.921171	0.014580
N	2.171697	0.508404	0.008024
N	1.657030	-1.661765	-0.004484
C	2.631840	-0.765073	-0.002696
O	-0.003343	2.649336	-0.010923
N	-3.027219	-0.818750	-0.072908
H	-3.235154	-1.681766	0.401230
H	-3.702212	-0.087710	0.082708
H	3.682838	-1.001149	-0.008297
H	2.709036	1.358871	0.011120
H	-0.990935	-2.299062	-0.147961

6

16

C	-1.680916	-0.460157	-0.002876
N	-0.684373	-1.369660	-0.009783
C	0.613131	-0.926285	0.000617
C	0.879898	0.461920	0.006744
C	-0.207387	1.309942	0.000770
N	-1.473258	0.835753	0.008675
N	2.243375	0.621640	0.008534
N	1.720613	-1.636051	-0.002581
C	2.669160	-0.631688	0.003178
O	-0.073720	2.631487	-0.005099
N	-2.966337	-0.924148	-0.062511
H	-3.161376	-1.819740	0.351623
H	-3.656973	-0.216611	0.123635
H	3.720705	-0.877967	0.002967
H	-0.963983	3.006142	-0.010302
H	-0.872073	-2.358859	-0.074069

6’

16

```
C -1.717334 -0.372671 -0.002996
N -0.769890 -1.339808 -0.009632
C  0.548664 -0.965085  0.000674
C  0.862308  0.403127  0.007216
C -0.173323  1.314110  0.000396
N -1.455395  0.914602  0.007420
N  2.226881  0.530489  0.008863
N  1.638449 -1.709378 -0.002600
C  2.617227 -0.739138  0.003195
O  0.022104  2.628853 -0.005373
N -3.023301 -0.772070 -0.060852
H -3.267351 -1.655915  0.352051
H -3.676069 -0.026594  0.115219
H  3.660675 -1.017348  0.002742
H -1.015471 -2.316035 -0.075580
H  0.978931  2.776160 -0.004750
```

7

16

```
C -1.727593 -0.386702  0.009171
N -0.727520 -1.346054  0.024625
C  0.546213 -0.837382 -0.007194
C  0.829010  0.500333  0.002431
C -0.260760  1.471012  0.000036
N -1.548785  0.884391  0.036252
N  2.198914  0.684285  0.010037
N  1.734929 -1.499900 -0.001889
C  2.705480 -0.513715  0.005319
O -0.124718  2.672741 -0.021711
N -2.998429 -0.913598 -0.078110
H -3.175518 -1.734440  0.479132
H -3.702218 -0.199762  0.029535
H  3.754206 -0.761026  0.001146
H  1.887443 -2.492914  0.015012
H -0.934032 -2.258934 -0.346114
```

8

```
16

C -1.632801 -0.593468  0.002763
N -0.693453 -1.476840  0.012265
C  0.576746 -0.978397 -0.011539
C  0.886494  0.431520  0.001313
C -0.149295  1.308205 -0.002006
N -1.413827  0.770030 -0.006053
N  2.247334  0.589649  0.018189
N  1.702989 -1.664704 -0.004876
C  2.654345 -0.669672  0.007171
O -0.025607  2.639104 -0.033304
N -2.965522 -0.957699 -0.067841
H -3.063237 -1.960215 -0.005411
H -3.577312 -0.481035  0.577594
H  3.705331 -0.921758  0.010491
H -0.822077  3.076408  0.284198
H -2.193427  1.361584 -0.248446
```

8’

```
16

C -1.665092 -0.532156 -0.001047
N -0.754292 -1.450730  0.006620
C  0.532666 -0.999847 -0.004414
C  0.876020  0.389485  0.011826
C -0.127088  1.302160  0.002593
N -1.401227  0.821855 -0.007456
N  2.236083  0.531918  0.016784
N  1.650508 -1.711023 -0.009594
C  2.620707 -0.740732  0.001896
O -0.023196  2.629575 -0.005592
N -3.004101 -0.860460 -0.070157
H -3.138181 -1.854611  0.035488
H -3.619972 -0.325537  0.522319
H  3.666457 -1.012934 -0.000855
H -2.156055  1.480769 -0.121203
H  0.921246  2.841336 -0.009520
```

9

16

```
C -1.715967 -0.446360 -0.005496
N -0.771672 -1.428249  0.002958
C  0.431560 -0.887155  0.000088
C  0.729049  0.459967 -0.006561
C -0.327156  1.449585  0.000038
N -1.569966  0.876691  0.001521
N  2.095687  0.576404 -0.004494
N  1.658340 -1.538771  0.005067
C  2.651657 -0.629708  0.001775
O -0.088656  2.655602  0.008046
N -2.999320 -0.901218 -0.055622
H -3.153156 -1.862205  0.188612
H -3.714774 -0.230522  0.158637
H  3.703656 -0.845482  0.002568
H  2.584058  1.459223 -0.006706
H  1.783120 -2.537806  0.007445
```

10

16

```
C -1.694989 -0.474064 -0.001364
N -0.697533 -1.340915  0.002468
C  0.614412 -0.872381  0.000850
C  0.930015  0.490761  0.002064
C -0.083896  1.478354  0.000965
N -1.412474  0.824646  0.005103
N  2.293935  0.591136  0.001774
N  1.693823 -1.634581  0.000651
C  2.679859 -0.679869  0.000785
O -0.093053  2.685317 -0.001252
N -2.984758 -0.901526 -0.063940
H -3.170570 -1.854263  0.199117
H -3.698964 -0.249746  0.211236
H  3.721391 -0.963651  0.000112
H -2.169534  1.492475 -0.018590
H -0.861253 -2.335481 -0.024052
```
